# Fast HPLC-MS/MS Method for Determining Penicillin Antibiotics in Infant Formulas Using Molecularly Imprinted Solid-Phase Extraction

**DOI:** 10.1155/2015/959675

**Published:** 2015-02-16

**Authors:** Mónica Díaz-Bao, Rocío Barreiro, José Manuel Miranda, Alberto Cepeda, Patricia Regal

**Affiliations:** Department of Analytical Chemistry, University of Santiago de Compostela, 27002 Lugo, Spain

## Abstract

The dairy cattle may suffer from different infections relatively often, but the inflammation of the mammary gland is very important to the farmer. These infections are frequently treated with penicillin antimicrobial drugs. However, their use may result in the presence of residues in animal products, such as milk powder and/or infant formulas, and it represents a potential risk for consumers. To monitor this, the EU has defined safe maximum residue limits (MRLs) through Commission Regulation (EU) number 37/2010. Although LC-MS is a trustful option for confirmation and quantification of antibiotics, the analysis of real samples with complex matrices frequently implies previous clean-up steps. In this work, precipitation polymerization has been used and different molecularly imprinted polymer (MIP) sorbents were tested and optimized for the fast and simultaneous solid-phase extraction (MISPE) of eight common penicillins (ampicillin, amoxicillin, oxacillin, penicillin G, penicillin V, cloxacillin, dicloxacillin, and nafcillin). The extracts were analyzed using liquid chromatography coupled to tandem mass spectrometry (LC-MS/MS) and the applicability of these polymers as sorbents for the extraction of penicillins at MRL levels in milk powder (infant formulas) was proved. The limits of detection and quantification were below the legal tolerances, except for LOQ for oxacillin and cloxacillin.

## 1. Introduction

The term antibiotic refers to a very diverse range of chemical substances that possess antibacterial activity. They can be either broad spectrum or narrow spectrum [1]. *β*-Lactam antibiotics (BLAs) constitute one of the most widely used antimicrobial drugs in veterinary medicine, especially to treat and prevent bacterial infections (respiratory, urinary, and mammary gland or skin infections) of dairy cattle. This group of antibiotics can be classified into several groups according to their structural characteristics: penicillins, cephalosporins, and, more recently, carbapenems. Their unique structural feature is the presence of the four-membered BLAs (2-azetidinone) ring [2].

**Figure 1:**
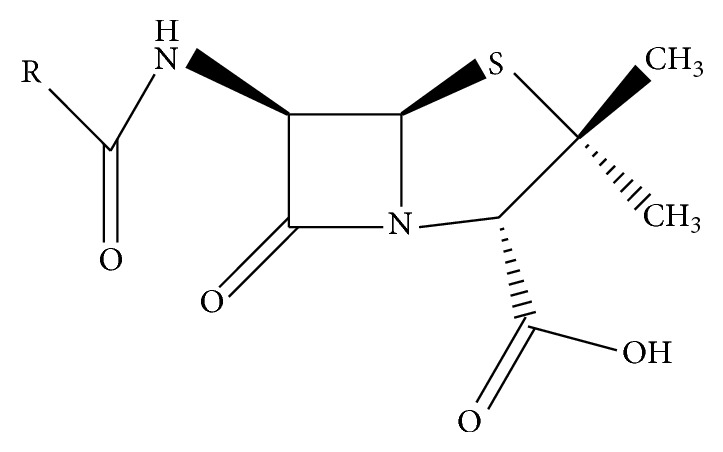
General structure of penicillins (R = lateral amino chain).

Penicillins are antimicrobial agents used against various microorganisms and exert their activity inhibiting the synthesis of the peptidoglycan layer of bacterial cell walls [1]. These molecules derive their activity from the 6-aminopenicillanic acid nucleus which is effective against mainly Gram-positive bacteria (Figure 1). Amoxicillin, ampicillin, penicillin G, penicillin V, cloxacillin, dicloxacillin, oxacillin, and nafcillin are registered drugs for the treatment of food-producing animals [3]. In dairy cattle, in addition to digestive and respiratory diseases, the inflammation of the mammary gland is very problematic for the farmer. Mastitis, although an animal welfare problem, is a big economic problem because it increases the somatic cell count, a milk quality indicator [4]. These infections are frequently treated with penicillins and they are also used for prevention on a regular basis. The incorrect use of these antibiotics may result in the presence of residues in edible tissues or derived products such as milk [5, 6]. It is well known that the improper use may have adverse effects on consumer health, including bacterial resistance to these drugs in humans, and also problems in dairy industry [7]. To avoid health risks for the consumer, the EU has defined safe maximum residue limits (MRL) for penicillins through Commission Regulation (EU) number 37/2010. The limits implemented in milk for penicillin antibiotics are shown in Table 1.

The determination of these compounds at their low MRL requires sensitive analytical methods to comply with current legislation. BLAs have poor stability in standard solutions and some specific compounds such as amoxicillin suffer degradation in solutions in one week of even fewer days [8]. For this reason, fast and straightforward methods of determination are generally preferred [9]. High performance liquid chromatography (HPLC) has been the most frequent technique for analyzing penicillins and other BLAs in different matrices, usually coupled with mass spectrometry (MS) [1, 3, 7, 10, 11]. Usually, this technique implies previous clean-up steps using common solid-phase extraction (SPE) procedures. The clean-up of the samples plays indeed a key role in determining the detection capability of the instrumental techniques because of its ability to reduce matrix interferences [12]. The main drawback of SPE techniques is the lack of selectivity of the sorbents. Molecularly imprinted polymers (MIP) are synthetic materials with recognition sites that specifically bind target molecules in mixtures with other compounds. MIP sorbents, which imitate natural recognition, are capable of meeting the demands of SPE and they may be reused several times with optimum recoveries [13]. The possible application of MIP as selective sorbents for the solid-phase extraction of some components of BLA group has been reported in recent years [12, 14–16]. During the development of MIP-based methods, it is interesting to develop polymers that allow the simultaneous detection of various analytes. The aim of the present work was testing the suitability of various MIP, synthesized using different templates and cross-linker monomers, for the simultaneous determination of eight penicillins (ampicillin, amoxicillin, oxacillin, penicillin G, penicillin V, cloxacillin, dicloxacillin, and nafcillin). The polymer was used as sorbent for the extraction of these antimicrobial drugs from milk and then followed by LC-MS/MS analysis. The effectiveness of these polymers was proved in spiked infant formulas at the level of interest (MRLs).

## 2. Materials and Methods

### 2.1. Chemicals and Reagents

Ampicillin (AMP), amoxicillin (AMX), oxacillin (OXA), penicillin G (PEN G), penicillin V (PEN V), cloxacillin (CLOX), dicloxacillin (DICLOX), nafcillin (NAFC), and piperacillin (PIPE) were obtained from Sigma-Aldrich (Madrid, Spain). The chemicals used for the polymers synthesis were methacrylic acid (MAA), divinylbenzene 80% (DVB-80), ethylene glycol dimethacrylate (EGDMA), trimethylolpropane trimethacrylate (TRIM), and the initiator 2,2′-azobis-(2-methylbutyronitrile) (AIMN) from Sigma-Aldrich. MAA, EGDMA, and TRIM were freed from stabilizers by distillation under reduced pressure and AIMN was recrystallized from methanol prior to use. Additionally, DVB was freed from stabilizers by passing through a small column packed with neutral alumina (Aldrich). HPLC grade solvents were supplied by Merck (Madrid, Spain).

### 2.2. Preparation of Polymers

The polymers were prepared by precipitation polymerization. OXA, AMX, and NAFC were used as template molecules and MAA was used as functional monomer. As cross-linkers DVB, EGDMA, and TRIM were tested, including different solvents in the polymerization mixture. All the polymers were prepared by mixing the template, functional monomer, and cross-linking monomer using a ratio of 1 : 6 : 20, under dilution conditions (4% wt.) in the porogen solvent and adding AIMN (2% wt. relative to monomers) as initiator. The different combinations are shown in Table 2. The polymerization mixtures were simultaneously introduced into a temperature controllable incubator equipped with a low-profile roller at 24 rpm and 60°C for 24 hours. The polymer particles were separated and cleaned by vacuum filtration through a nylon membrane filter of 0.45 *µ*m of pore diameter, using 50 mL of acetonitrile and 50 mL of methanol. Then the imprint molecule was removed by Soxhlet extraction for 8 h using a methanol/acetic acid mixture (1 : 1). In each case, nonimprinted polymers (NIP) were prepared in the same way but without the addition of template.

### 2.3. LC-MS/MS Analysis

The obtained recoveries using different polymers were calculated using an HPLC-MS/MS method. Separation was performed in a 1100 series HPLC system from Agilent Technologies (Minnesota, USA). A Synergi 4 *µ*m MAX-RP 80A (100 × 2 mm) column from Phenomenex (Torrance, CA, USA) was used. The mobile phase was acetonitrile (A) mixed on a gradient mode with 0.2% aqueous formic acid (B) at a flow rate of 300 *µ*L min^−1^. After the first 2 minutes with very aqueous mobile phase at 90% (B), binary gradient mixing was initiated as follows: (B) 90% to 0% for 16 min and 0% to 90% again for 3 min; at this point the gradient was kept isocratic for 4 min. The obtained chromatographic retention times (RT) are included in Table 3. A Q-Trap 2000 mass spectrometer with ESI Source from AB SCIEX (Toronto, Canada) was used, working in positive mode. For quantification, the most intense multiple reaction monitoring (MRM) transition was monitored along with a second transition for qualitative confirmation (Table 3).

### 2.4. Optimization of Molecularly Imprinted Solid-Phase Extraction (MISPE)

Molecularly imprinted and nonimprinted polymers (0.05 g) were placed in empty SPE glass cartridges. The cartridges were coupled to an SPE manifold and several experiments were carried out loading 1 *µ*g of each analyte in 1 mL of different loading solvents (acetonitrile and toluene) and using 6 mL of various washing solutions (acetonitrile, methanol, and toluene with different % of acetonitrile). In parallel, the same experiments were carried out on NIP cartridges in order to prove the existence of template-specific imprinted sites into the MIP. The obtained elution (3 mL of methanol 1% acetic acid) was evaporated under nitrogen stream and redissolved in 1 mL of mobile phase for HPLC-MS/MS analysis. Recovery values were calculated using a standard calibration curve.

### 2.5. MISPE of Infant Formulas

After MISPE optimization, infant formulas (0.3 g) spiked at the level of interest (MRL, Table 1) with a mixture of the selected penicillins in acetonitrile were extracted using the selected polymer (NAFC-MAA-EGDMA-ACN-AIMN) and the optimized loading-washing-elution conditions. Thus, the MISPE protocol was acetonitrile-acetonitrile-methanol 1% acetic acid. Samples were diluted with 1 mL of acetonitrile, vortexed for 1 min, and centrifuged at 5,000 g for 10 min and the supernatant was directly loaded into the selected MIP cartridge. The column was washed with 6 × 1 mL acetonitrile and the elution was performed with 3 × 1 mL of elution solvent acidified methanol (1% acetic acid). After washing and eluting, the extract was dried under a nitrogen stream at 30°C and redissolved in 100 *µ*L of mobile phase B. Thirty microliters of extract were immediately injected into the chromatographic system and assayed with the developed HPLC-MS/MS method.

## 3. Results and Discussion

### 3.1. Preparation of Polymers and MISPE Optimization

The used polymerization technique was precipitation polymerization, which yields an increased rebinding capacity and more homogeneous distribution binding sites with microspherical shapes of uniform sizes. In this study, oxacillin, amoxicillin, and nafcillin were selected as representative structures of the penicillin group and possible adequate templates to synthesize MIP sorbents that would be effective in extracting the selected analytes. These templates were combined with MAA and DVB, EGDMA, and/or TRIM, in different porogen solutions. After polymerization, the prepared MISPE cartridges were tested using standard solutions of the selected penicillins. The eligibility criterion in terms of recovery requirements to select the best polymer was set at a minimum of 50% recovery percentage for all the selected analytes.  Table 4 shows the maximum achieved recoveries for each polymer, obtained during the optimization experiments (different loading and washing steps; elution was always with methanol 1% acetic acid).

In general, recoveries with OXA- and/or AMX-based MIP were lower than those with the NAFC-based polymers. These polymers provided good retention of penicillins in preliminary experiments using acetonitrile as loading solvent. The acetonitrile was evaporated after the sorption step (pass-through the cartridges) and redissolved in mobile phase for HPLC analysis, proving that approximately 90% of most penicillins were retained in the MIP. However, the analytes would elute during the washing steps (using acetonitrile or methanol), obtaining no more than 30% of recovery during elution. For this reason, additional experiments were performed with toluene, in both loading and washing steps, and also using toluene with different percentages of acetonitrile (5, 10, and 20%) as washing solution. Even then, the elution was not homogeneous for all penicillins, getting good recoveries in some cases (OXA and PEN G with OXA-based polymer) but not for the whole group of antimicrobials. NAFC was a more adequate template to obtain polymers and its combination with MAA, EGDMA, and acetonitrile as porogen solvent permitted to obtain MIP sorbent for the simultaneous extraction of all the selected analytes (Table 4). A more homogeneous recovery for all penicillins was preferred according to the low MRL of these analytes in milk. Additionally, this polymer provided the higher differences (>30%) between MIP and NIP cartridges. Also, some polymerization problems were found when using OXA and/or AMX as templates in combination with DVB in acetonitrile/toluene porogen, mainly due to the low solubility of these drugs in nonpolar solvents (the mixture ACN/TOL presents the lower polarity of all the tested porogens). In this case, no polymerization was achieved, because it was not possible to dissolve the template in the polymerization mixture, which is a key factor in MIP synthesis. When using DVB as cross-linker, the combination of acetonitrile and toluene is required to obtain polymers with good performance and with the production of monodisperse, imprinted-polymer beads with well-developed, permanent pore structures [13].

Summing up, NAFC-MAA-EGDMA-ACN was selected as SPE sorbent because it provided the higher retention capacity for the eight penicillins studied (ampicillin, amoxicillin, oxacillin, penicillin G, penicillin V, cloxacillin, dicloxacillin, and nafcillin), enabling the simultaneous extraction of these drugs at the level of interest in milk. Therefore, this polymer was selected for further investigations and validation with real samples. After optimizing the MISPE conditions, an adequate and detectable amount of PIPE was added to the samples to act as internal standard.

### 3.2. MISPE Application to Infant Formulas and Analytical Performance

The developed procedure with NAFC-based MIP was applied for the determination of penicillin residues in infant formulas, as representative milk powder samples. Acetonitrile as loading and washing solvent provided clean extracts with satisfactory recoveries for all penicillins in real samples (≥60%), as it is shown in Table 5. In 2010, Yin et al. hinted the suitability of nafcillin as template, using this compound as pseudo-template to develop a MISPE protocol for selective screening of other four penicillins in water, a much less complex aqueous matrix [14]. In this study, the template was also determined and no measurable bleeding of nafcillin was detected in the blank samples. Apart from nafcillin, seven different penicillin drugs were extracted with this polymeric sorbent and determined by LC-MS/MS. The matrix was selected based on the importance that residues of antimicrobials in milk may have for vulnerable population, as babies and children. Powdered infant formulas were sampled as they were liquid formulas because there is no MRL specifically established for powdered formulas. Thus, a sample of 0.3 g of powder formula was analyzed as it is the amount necessary to obtain 2 mL of liquid formula, according to manufacturer's directions. The sample size was also selected based on the instrumental detection limits.

The analytical MISPE procedure was fast, as it only included a simple protein precipitation with acetonitrile, followed by the direct loading of the extract into the NAFC-MAA-EGDMA-ACN polymeric SPE cartridge. Protein precipitation was necessary to avoid the interference of macromolecules present in milk with the active imprinting sites [16]. The total clean-up time was always less than 20 minutes. Usually, the determination of penicillins in milk is included in multiresidue and multiclass methods, implying various purification steps such as defatting, centrifugation, dilution, and less selective SPE protocols [1, 3, 7, 10, 11]. Thus, the present experiments were considered successful, as the goal of the study was to develop a fast, reusable, affordable, and simple method for the analysis of these drugs in milk. The obtained recoveries were similar to or lower than those obtained by Ghidini et al., who used only sample (acidic) precipitation, centrifugation, and filtration. However, they used a higher initial sample amount and injected a higher volume of the final extract [9].

The selected polymer was chosen basing on the previous recovery experiments and aiming at a reasonable recovery (compromise between matrix effects and low MRLs) for all the analytes. The recovery came out at between 60 and 91%, similar values to those recently obtained for some of the compounds by Giovannoli et al., who used silica beads as support for imprinting and applied the method to milk samples [12]. These authors prepared silica-imprinted beads using 6-aminopenicillanic acid as mimic template. The imprinted beads were used to extract penicillin V, nafcillin, oxacillin, cloxacillin, and dicloxacillin in milk at MRL levels, and the extracts were analyzed using electrokinetic chromatography. The possible use of magnetic molecularly imprinted polymers (MMIP) to selectively separate some *β*-lactam antibiotics from milk has been reported [15], using also MAA as functional monomer and EGDMA as cross-linking agent. However, in that previous study, the selected template molecule was penicillin V and the magnetic MIP sorbent was applied to determine only PEN V, AMX, and OXA in milk samples. The recoveries and detection limits were similar to those obtained in this study, using a less complicated approach.

**Figure 2:**
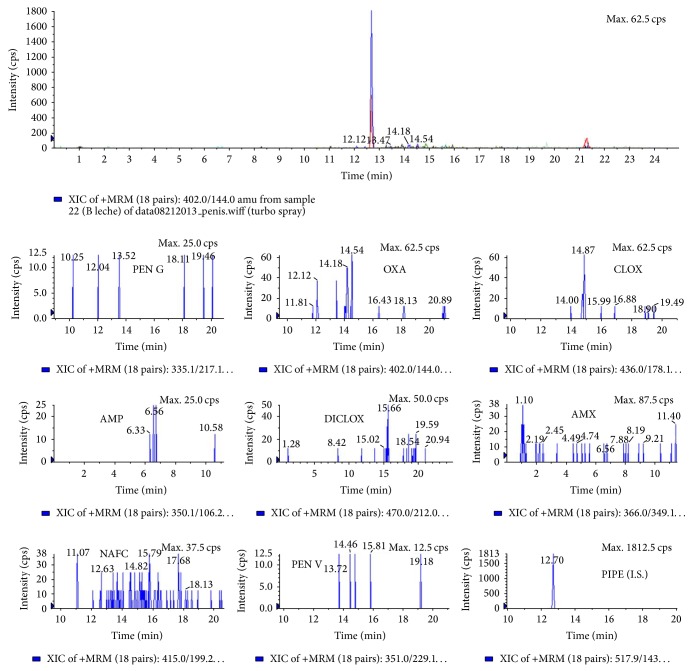
Chromatogram of a blank infant formula (internal standard: piperacillin).

**Figure 3:**
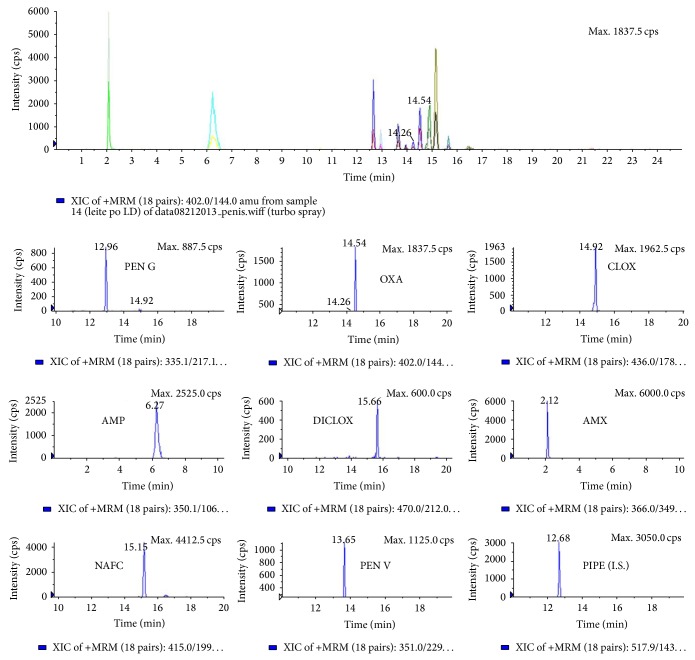
Chromatogram of an infant formula spiked at the MRL levels for ampicillin, amoxicillin, oxacillin, penicillin G, penicillin V, cloxacillin, dicloxacillin, and nafcillin (internal standard: piperacillin).

As an example of successful clean-up, Figure 2 shows a chromatogram of a blank sample and Figure 3 shows a chromatogram obtained during the analysis of a spiked infant formula, containing 4 *μ*g L^−1^ of AMX, PEN G, PEN V, and AMP and 30 *μ*g L^−1^ of OXA, CLOX, DICLOX, and NAFC (MRLs). No interfering peaks could be observed in the chromatogram. The obtained recoveries, limit of detection (LOD), and limit of quantitation (LOQ) for each antibiotic are summarized in Table 5. It is clear that the detection limits achieved by the developed procedure are low enough to allow the analysis of penicillins in milk/milk powder samples at real concentration levels, except for the LOQs for OXA and CLOX. Respectively, LOD and LOQ values were expressed as the concentration of the target analyte that produced a chromatographic signal response, three and ten times higher than the chromatographic baseline or background noise surrounding that peak. The linearity of the method was evaluated by preparing blank and spiked samples (at MRL and 1.5, 2, and 3 times the MRL concentration). Calibration curves were constructed by plotting the concentration against area ratio (analyte peak area/internal standard peak area). Linearity coefficients ranged from 0.95 to 0.99. Precision was expressed as intra- and interday precision (%CV), from five repeated experiments performed at two times during the same day (ten injections) and on three consecutive working days (fifteen injections). Precision is concentration dependent and acceptable precision at the lower concentrations was set at 20% (Table 5). If highly contaminated samples need to be analyzed, the recommendation is diluting the samples previous MISPE and/or preparing MISPE cartridges with more polymeric content. Otherwise, the imprinting sites would be saturated and the analytical signal would reach a maximum or even decrease.

## 4. Conclusions

Various imprinted polymers have been prepared to obtain a MIP-based material with proper characteristics to be used as selective MISPE sorbent for the fast and simultaneous extraction of eight important penicillins in milk/milk powder. Amongst the different combinations tested, only the MIP prepared in acetonitrile using nafcillin as template molecule and EGDMA as cross-linker monomer showed a reasonable recovery for all the selected analytes. The latest fact proves that testing various assays/polymeric combinations is preferable when designing MIP for solid-phase extraction, especially in the case of a group of structurally related compounds.

From the observed data it may be concluded that this NAFC-based MIP sorbent is suitable for the extraction of penicillin drugs from milk powder. Molecularly imprinting technologies provide an alternative solution for SPE, allowing a simple and straightforward clean-up strategy. With the developed MISPE protocol, enough recovery was achieved for eight common penicillin antibiotics at their low level of interest (MRL). The combination of this fast extraction approach with LC-MS/MS determination resulted in an affordable analytical method with good performance in terms of linearity and precision.

## Figures and Tables

**Table 1:** MRLs established by Commission Regulation (EU) number 37/2010 for penicillins residues in milk.

Analyte	MRL (*µ*g·kg^−1^)
Oxacillin	30
Cloxacillin	30
Dicloxacillin	30
Nafcillin	30
Penicillin G	4
Penicillin V	4
Ampicillin	4
Amoxicillin	4

**Table 2:** Composition of different MIP synthesized for the simultaneous extraction of penicillin drugs.

Template	Functional monomer	Cross-linkers	Porogens	Initiator	Polymerization problems
Oxacillin		DVB	ACN/TOL	AIMN	Not dissolved
		EGDMA	MeOH	AIMN	
Amoxicillin		DVB	ACN/TOL	AIMN	Not dissolved
		EGDMA	MeOH	AIMN	
Nafcillin	MAA	DVB	ACN/TOL	AIMN	
		TRIM	ACN	AIMN	
		TRIM	MeOH	AIMN	
		**EGDMA**	**ACN**	**AIMN**	
		EGDMA	MeOH	AIMN	

MAA: methacrylic acid; EGDMA: ethylene glycol dimethacrylate; DVB: divinylbenzene; AIMN: 2,2′-azobis-(2-methylbutyronitrile); TRIM: trimethylolpropane trimethacrylate; MeOH: methanol; ACN: acetonitrile; TOL: toluene.

**Table 3:** MRM transitions of each analyte and collision energy (CE) for mass spectrometry detection and chromatographic retention time.

Compound	Mw	Precursor ion	Fragment ion	CE (volts)^*^	RT
Amoxicillin	365.4	366	349	13	2.1
			114	25	

Oxacillin	401.4	402	144	33	14.5
			186	21	

Cloxacillin	435.9	436	178	31	14.9
			220	21	

Penicillin G	334.4	335	217	17	12.9
			202	31	

Penicillin V	350.4	351	229	19	13.7
			257	17	

Ampicillin	349.4	350	106	21	6.4
			160	9	

Dicloxacillin	470.3	470	212	33	15.7
			254	23	

Nafcillin	414.4	415	199	17	15.2
			171	51	

^*^CE: collision energy in volts.

**Table 4:** Maximum recoveries achieved for the different penicillin-based MIP sorbents during MISPE optimization using standard solutions.

MIP^a^	Recovery (%)							
	AMX	OXA	CLOX	PEN G	PEN V	AMP	DICLOX	NAFC
OXA-MAA-EGDMA-MeOH^b^	45	60	28	77	34	50	25	36
AMX-MAA-EGDMA-MeOH^b^	15	10	10	6	7	10	7	8
NAFC-MAA-DVB-ACN/TOL^c^	27	31	27	22	35	45	22	50
NAFC-MAA-TRIM-ACN^c^	25	65	66	54	62	45	58	56
NAFC-MAA-TRIM-MeOH^c^	22	82	61	54	65	38	57	51
NAFC-MAA-EGDMA-ACN^c^	58	88	82	56	70	65	63	77
NAFC-MAA-EGDMA-MeOH^c^	20	58	43	42	56	40	51	49

^a^Polymerization mixture, that is, template-functional monomer-cross-linking monomer-porogen (initiator was always AIMN). ^b,c^Optimized extraction conditions for maximum recoveries, elution always with methanol 1% acetic acid; ^b^loading: toluene; washing: toluene 10%; ^c^loading and washing: acetonitrile.

**Table 5:** Mean recoveries (ten samples), precision, limit of detection (LOD), and limit of quantification (LOQ) obtained for the determination of penicillin drugs in milk powder (infant formula) using NAFC-MAA-EGDMA-ACN polymer as MISPE sorbent, in combination with HPLC-MS/MS.

Parameter^a^	Analyte							
	AMX	OXA	CLOX	PEN G	PEN V	AMP	DICLOX	NAFC
Recovery (%)	60	84	91	60	60	66	62	74
CVr (%)	9.2	12.1	3.6	5.5	13.9	10.3	1.4	8.1
CVR (%)	10.7	19.2	19.3	9.9	16.0	12.9	2.4	11.1
LOD (*µ*g kg^−1^)	0.9	23.6	15.5	0.7	1.2	1.1	1.4	7.4
LOQ (*µ*g kg^−1^)	3.1	78.4	51.6	2.4	3.9	3.6	4.8	24.6

^a^CVr: repeatability, greater intraday CV%; CVR: within-lab reproducibility, greater interday CV%.
